# Behavioral Response of Invertebrates to Experimental Simulation of Pre-Seismic Chemical Changes

**DOI:** 10.3390/ani5020206

**Published:** 2015-03-31

**Authors:** Rachel A. Grant, Hilary Conlan

**Affiliations:** 1 Department of Animal and Land Sciences, Hartpury College, Hartpury, Gloucester GL19 3BE, UK; 2 Department of Life Sciences, Anglia Ruskin University, East Rd, Cambridge CB1 1PT, UK; E-Mail: hilary.conlan@anglia.ac.uk

**Keywords:** earthquakes, animal behavior, *Daphnia pulex*, earthworms, hydrogen peroxide, positive holes

## Abstract

**Simple Summary:**

We exposed two invertebrates to hydrogen peroxide to mimic some of the conditions that occur before large earthquakes. Water fleas changed their position in an aquarium to avoid the hydrogen peroxide but earthworms appeared not to be affected and did not change position. We discuss this in the context of unusual animal behavior often seen before earthquakes.

**Abstract:**

Unusual behavior before earthquakes has been reported for millennia but no plausible mechanism has been identified. One possible way in which animals could be affected by pre-earthquake processes is via stress activated positive holes leading to the formation of hydrogen peroxide at the rock water interface. Aquatic and fossorial animals could be irritated by H_2_O_2_ and move down the concentration gradient. Here, we carry out avoidance tests with hydrogen peroxide in two model organisms; *Daphnia pulex* and earthworms. *Daphnia* were found to move away from increasing concentrations of H_2_O_2_ but earthworms appeared unaffected. It is possible that earthworm swarming behavior, reported frequently before earthquakes, is caused by electric field shifts or another unknown mechanism, whereas zooplankton may be affected by increasing levels of H_2_O_2_.

## 1. Introduction

Unusual behavior prior to earthquakes has been reported for millennia [1], although most reports are classified as anecdotal and usually consist of *post hoc* recollections by local people. Nevertheless, there are similarities between reports, which, although the seismic events differ spatially and temporally, report similar behavior in animals at similar distances and times relative to earthquakes [2]. Anecdotal reports of unusually large numbers of earthworms (*Lumbricus terrestris*) appearing on the surface of the ground prior to seismic events are common [1,3].

There are also some systematic evaluations of animal behavior changes prior to earthquakes for which behavior has been recorded in a methodical way. One particular case concerns the unusual behavioral changes in mating common toads (*Bufo bufo*) a few days before the L’Aquila M = 6.3 earthquake in 2009, and a subsequent evaluation based on water chemistry changes [4,5]. Common toads normally breed once a year in an “explosive” manner—the breeding occurs over a three-week period, and it is highly unusual for breeding to be interrupted once it has started. However five days prior to the L’Aquila earthquake, in the middle of the reproductive season, all toads disappeared from their breeding site, only reappearing after the earthquake was over [4]. A subsequent analysis [5] of the weather data for the site revealed that the site was heavily waterlogged. It has been reported that before many earthquakes, water chemistry is altered [6,7] and Grant *et al.* [5] report on spectroscopic changes in water chemistry along the North Anatolian Fault Zone (NAF) in Turkey before the M = 7.6 Izmit earthquake of August 17, 1999, showing alterations in emission spectra prior to earthquakes in the 340 nm range. Although the exact nature of the chemical changes are, as of yet, unknown, they may relate to oxidation of organic matter by H_2_O_2_ [5]. Research has shown that some zooplankton appear to be sensitive to environmental changes relating to earthquakes [8] and the water flea, *Daphnia spp* is particularly sensitive to water quality and is used to test water cleanliness. Grant *et al.* [5] proposed that animals’ behavior changes before earthquakes may not be an evolved response but simply an avoidance reaction to aversive stimuli resulting from geophysical processes occurring in the earthquake preparation zone. If this is the case then animals are not anticipating seismic activity but merely distancing themselves from unpleasant or harmful substances in their environment. Based on these observations, we considered that earthworms and *Daphnia pulex* (a small planktonic crustacean) would make satisfactory model organisms for the study of chemical changes occurring prior to earthquakes.

**Figure 1:**
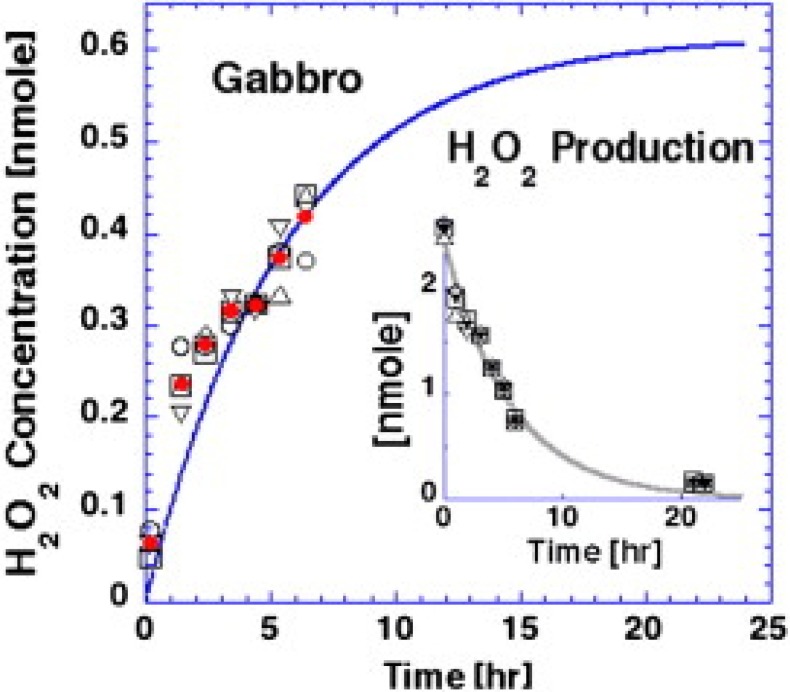
Showing the formation of H_2_O_2_ in a pool above slices of Gabbro placed under mechanical stress in laboratory conditions. After Balk *et al.* [9].

There exist dormant defects within igneous and high-grade metamorphic rocks which, when stressed, release electronic charge carriers. These charge carriers are defect electrons associated with O^−^ in a matrix of O^2^ and called positive holes (pholes) and they are able to travel rapidly and far through the rock, in the order of meters in the laboratory and km in the field [9,10]. These cause a range of follow-on reactions when they arrive at the Earth’s surface, primarily air ionization and at the rock-water interface oxidation of water to hydrogen peroxide [9,10]. The formation of hydrogen peroxide at the rock water interface resulting from stressed rock has been demonstrated by Balk *et al.* [9] in the laboratory. Slices of Gabbro (an igneous rock, formed from cooling magma which underlies most of the Earth’s crust), were placed under mechanical stress underneath a small chamber containing water. H_2_O_2_ was formed in the chamber above the rock (Figure 1).

It has been suggested [5] that the hydrogen peroxide (and subsequent oxidation products) could cause avoidance reactions in animals which are in intimate contact with the substrate, and that this may be a possible mechanism for unusual pre-seismic behavior in aquatic or burrowing animals. Although hydrogen peroxide has not been measured in field conditions in connection with earthquake preparation zones, massive amounts of positive air ions have been recorded by air ionization sensors set up alongside the QuakeFinder stations along the San Andreas Fault system in California and along the subduction zone in Southern Peru [8,9,10]. Where these ions come into contact with water, it can be expected that large amounts of hydrogen peroxide will be formed. As earthquake preparation zones can last for weeks or months, a continuous stream of ions will lead to the continuous formation of H_2_O_2_. Although H_2_O_2_ is short-lived, as it either disassociates to form water and oxygen, or reacts with available molecules, it is likely that animals in contact with the substrate, such as earthworms, or aquatic animals, may encounter H_2_O_2_. Massive ionization leading to the formation of H_2_O_2_ was postulated as one possible cause of toads leaving their breeding site before the L’Aquila earthquake [5]. Temporally co-incident disturbances in the ionosphere detected using radio-sounding methods, support the idea of massive air ionization in the earthquake preparation zone around L’Aquila. We aimed to test the hypothesis that hydrogen peroxide will cause an avoidance reaction in two model experimental organisms; *Daphnia* spp, and earthworms.

## 2. Experimental Section

### 2.1. Earthworm Perfusion Test

**Figure 2:**
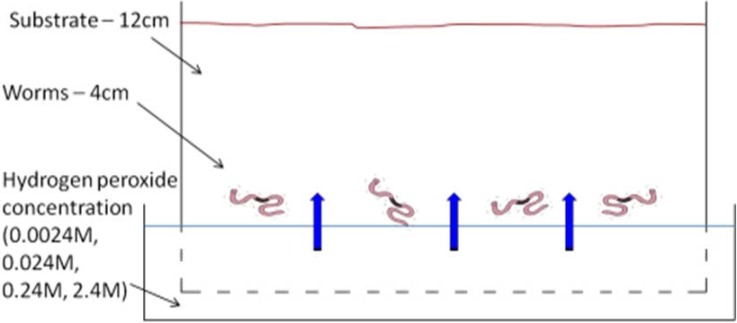
Showing the earthworm perfusion avoidance test.

Mature earthworms, *L. terestris*, (10–14 cm) were kept in a standard substrate (John Innes No 3 potting compost) for one week prior to the experiment. Only active earthworms were used. The substrate was air dried (mean 10% soil moisture reading). Clear plastic containers (22 × 48 cm) with perforated bases were filled with the prepared substrate to a depth of 12 cm (Figure 2). Four earthworms were used for each treatment and each worm was only used once. Worms were randomly selected from group of 200 all living together in the same conditions. Four earth worms were spaced out evenly at a depth of 9 cm. The containers were lowered into a 5 cm deep water-bath with varying concentrations of hydrogen peroxide: zero (control), 0.0024 M, 0.024 M, 0.24 M, and 2.4 M prepared with degassed tap water. Each experiment was replicated three times (using 12 earthworms per treatment in total), for a total of 60 earthworms in this part of the experiment. The depth of the earthworms was recorded after 1 hour and 24 hours. Video recording was carried out to record any earthworms coming to the surface. The mean soil moisture readings after 24 hours at a depth of 3 cm was 24% and at 9 cm was 69%.

### 2.2. Earthworm Pre-Mixed Soil Test

The earthworms were sampled as above (4 worms, 3 replicates and 5 treatments, with worms only being used once, for a total of 60 worms). Concentrations of hydrogen peroxide (zero (control) 0.0024 M, 0.024 M, 0.24 M and 2.4 M) were prepared with 4 liters of degassed tap water. Each concentration was mixed with air dried John Innes No 3 potting compost to give a mean soil moisture reading 30%. Earthworm movements within the soil were recorded as above.

### 2.3. Daphnia Test

**Figure 3:**
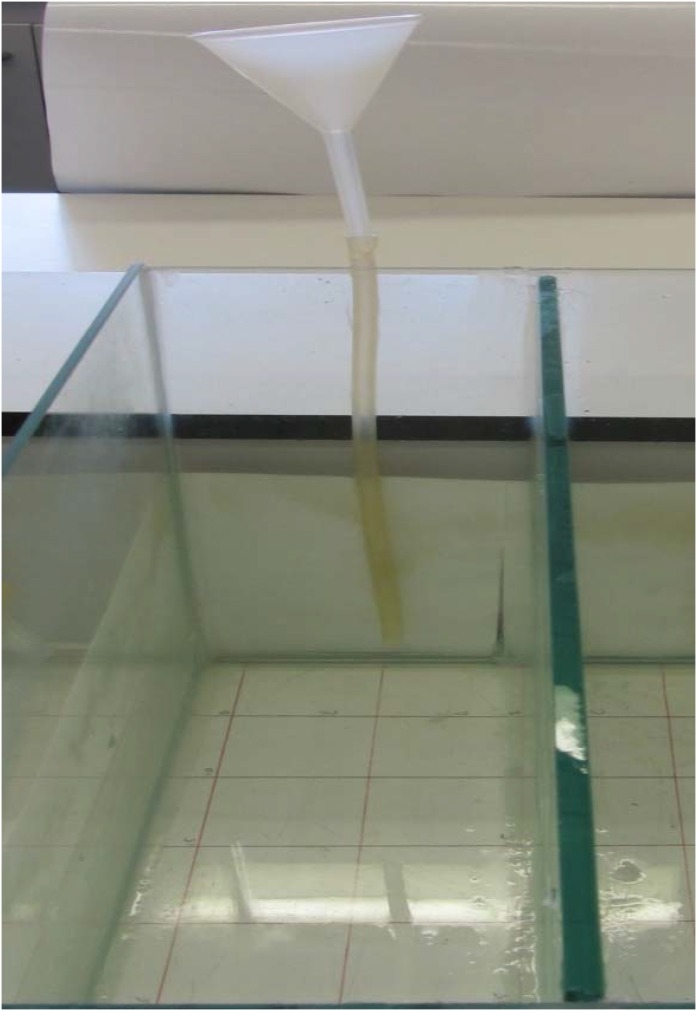
Showing the glass tank used for the *Daphnia* avoidance test.

**Figure 4:**
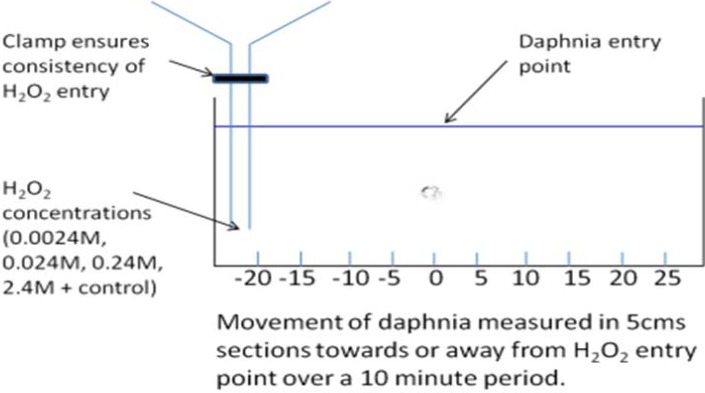
Showing the setup for the *Daphnia* avoidance test.

Opaque glass tanks (11 × 48 cm) were filled with four liters of degassed tap water (Figure 3 and Figure 4). A location grid of 5 cm squares was placed under each tank. The entry point was marked as zero with filling point at −20 cm. A standard filling procedure was developed to ensure that the hydrogen peroxide added would diffuse long the length of the tank in 10 minutes, allowing a concentration gradient to be established but without creating significant water movement. Concentrations of hydrogen peroxide in 200 mL were prepared to give a final concentration of zero (control), 0.0024 M, 0.024 M, 0.24 M, and 2.4 M in four liters.

One minute after the filling procedure, pairs of *Daphnia* were introduced to the center of the tank and their position recorded every 30 seconds in relation to the entry point for 10 minutes. Control experiments with tap water demonstrated that the recorded movement was not affected by the filling procedure or water movement. After 10 minutes the final position was recorded. Each experiment was replicated ten times.

## 3. Results and Discussion

### 3.1. Earthworm Perfusion Test

The earthworms did not change position with increasing concentrations of H_2_O_2_ within the first hour. After 24 hours there was no statistical difference in the positions of the earthworms (ANOVA: F = 0.72; df = 4; *p* = 0.58). No surface movement was seen throughout the 24 hours. No earthworms died in the experiment.

### 3.2. Earthworm Pre-Mixed Soil Test

The earthworms did not change position with increasing concentrations of H_2_O_2_ within the first hour. After 24 hours there was no statistical difference in the positions of the earthworms (ANOVA F = −0.45; df = 4; *p* = 0.77). Two worms were recorded coming to the surface (6 hours 45 minutes - H_2_0_2_ 0.024 M and 15 hours 30 minutes - H_2_0_2_ 0.024 M). Neither worm stayed on the surface longer than 15 minutes. No earthworms died in the experiment.

### 3.3. Daphnia Test

**Figure 5:**
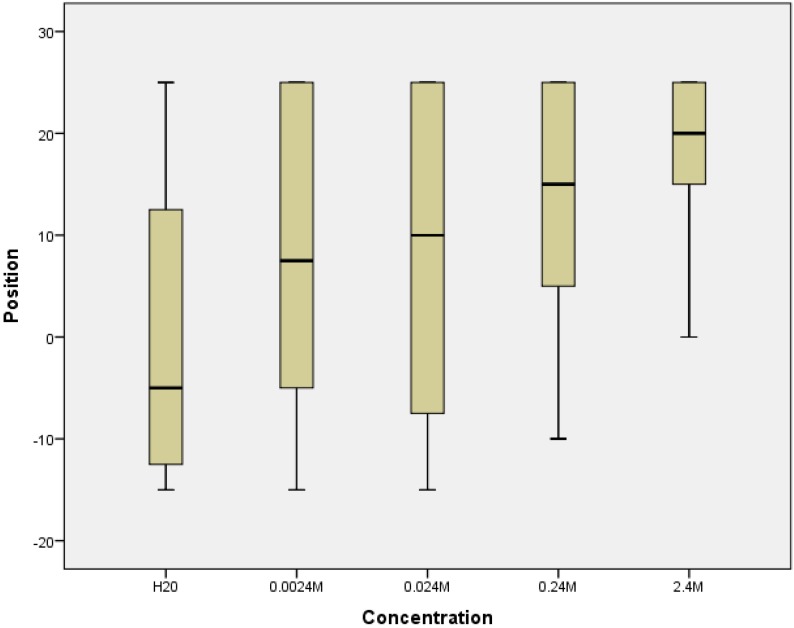
The mean position (based on 10 replicates of two *Daphnia*) (±standard deviation) of the *Daphnia* 10 minutes after varying concentrations of hydrogen peroxide was added to the tank. Start position at 0, −20 cm = entry point of hydrogen peroxide, 30 cm = furthest distance from entry point of hydrogen peroxide.

The position of the *Daphnia* after ten minutes varied significantly with increasing concentrations of hydrogen peroxide, with the average position being further from start point with increasing concentrations of H_2_O_2_ (Kruskal-Wallis: X^2^ = 15.895, n_1–5_ = 20, P = 0.003) (Figure 5). The death rate of the *Daphnia* (which was less than 3% overall) was not affected by the varying concentrations of H_2_O_2_ after 10 minutes.

## 4. Conclusions

*Daphnia* appear to be showing a simple avoidance reaction to the presence of H_2_O_2_ at 0.24 M and above. It is overly speculative to extrapolate these results to complex higher organisms such as the toads at L’Aquila. However, the results may support the hypothesis of Grant *et al.* [5] that one of the causes of avoidance reactions some animals before earthquakes may be the release of H_2_O_2_ into water. These results are preliminary and further investigations into the effects of the particular type of water chemistry changes which may occur in the earthquake preparation zone on aquatic organisms should be carried out. The concentration of hydrogen peroxide necessary to elicit a response was quite high, higher than the concentration shown to be formed in laboratory experiments [9], however the area of stressed rock in the field would be much greater and the positive ions are known to be released in massive amounts. Although H_2_O_2_ is a transient molecule, in the earthquake preparation zone a massive and continuous stream of ionization would be expected, meaning that H_2_O_2_ concentrations in the field may reach levels much higher than those shown in the laboratory experiment where only a 60 × 7.5 × 7.5 cm^3^ block of Gabbro was used. Earthworms did not appear to be affected by increasing concentrations of H_2_O_2_ in either the pre mixed soil or H_2_O_2_-infused soil. This could be for several reasons:

1. Worms are not affected by H_2_O_2_ at the concentration that we used,
2. The H_2_O_2_ could be decomposed more rapidly in soils than in water due to surface area effects,
3. The higher presence of organic matter in soils may provide numerous oxidation pathways for the H_2_O_2_,
4. There could be behavioral confounding factors due to handling the worms.

These results could imply that earthworm behavior before earthquakes could be due to a factor other than chemical composition of the soil. Ikeya [11] showed that earthworms respond to electric fields by swarming. Placing electrodes into the soil and generating current is a standard method of sampling earthworms as it causes them to rise to the surface [12]. Electromagnetic changes are common in the earthquake preparation zone and have been reported before many large earthquakes [13]. It has been speculated that animal behavior anomalies, including earthworm swarms could be due to EM shifts [3]. While our paper does not provide evidence that earthworms may be more affected by EM variations than soil chemistry anomalies, it paves the way for further investigation.
